# Letter to the editor regarding “spatiotemporal association of low birth weight with Cs-137 deposition at the prefecture level in Japan after the Fukushima nuclear power plant accidents”

**DOI:** 10.1186/s12940-020-00651-5

**Published:** 2020-11-25

**Authors:** Sani Rachman Soleman, Tomoko Fujitani, Kouji H. Harada

**Affiliations:** 1 Department of Health and Environmental Sciences, Kyoto University Graduate School of Medicine, Yoshida Konoe, Sakyo, Kyoto, 6068501 Japan; 2 Department of Public Health, Faculty of Medicine, Universitas Islam Indonesia, Yogyakarta, 55584 Indonesia

**Keywords:** Low birth weight, Radiation, Epidemiology, Statistics, Ecological study, Fukushima nuclear disaster

## Abstract

In the previous report, association between increased low birth weight prevalence and radiocesium deposition after 2011 Fukushima nuclear disaster was found. However, the statistical analyses therein raised several questions. First, ecological variables are not justified enough to adjust potential confounding. Second, the spatiotemporal regression model does not consider temporal reduction in radiation dose rate. Third, dose-response plot between dose rates and odds ratios overestimates R^2^ and underestimates *p* value.

**Dear Editor**

Regarding with a recent study on Cs-137 exposure with low birth weight (LBW) in infants in Japan by Scherb and Hayashi [1], we express concerns on data analyses, particularly in Figures 4 and 5 of the article.

Firstly, LBW can be influenced by various individual factors such as body weight change of mothers during pregnancy, smoking, etc. The authors included spatiotemporal effects in logistic regression model (but details of modeling were not provided), and analyzed data without individual factors. Authors included ecological variables, but they are not enough to control individual variations for LBW. How can annual population, number of physicians adjust the potential risk factors for LBW? In addition, if triple disasters affected those background factors, OR jump could be observed. Hence, individual risk factors are essential for the analysis because the observed OR for Cs-137 is relatively small and easily influenced by confounding.

Secondly, Figure 4 of the article claimed ionizing radiation increased the prevalence of LBW. The logistic model assumed the level-shift after 2011 persists to 2018 at same magnitudes. According to previously study [2], air radiation dose rate from deposited fallout was highest at initial time of deposition, but the dose decreased to less than 40% only by physical decay until 2016. Further, migration of radionuclide in soil reduced the dose rate to 20% of initial rate [2]. Thus, it is unlikely that the effect on Cs-137 persisted for years without any attenuation. In other words, observed OR jump can be attributable to other factors in triple disasters or regional trends.

Thirdly, we found an overestimation of R^2^ and underestimation of *p* value of the regression in Figure 5 of the article. Authors mixed data of 37 prefectures into one point, and made variations of them small. As shown in Tables 2 and 3 of the article, even in non-contaminated regions showed high odds ratios over 1.1. Furthermore, authors analyzed them with restricted linear regression model (intercept was fixed to 1), but we examined them in unrestricted regression that showed R^2^ of 0.37 and *p* = 0.046. The R^2^ was further decreased to 0.11 in the analysis of original 47 records (Fig. 1 in this letter). Authors intended to ‘avoid an overly scattered picture’, but the manipulation caused overestimation of R^2^. In addition, there was no rationale to employ restricted linear regression analysis.

**Fig. 1:**
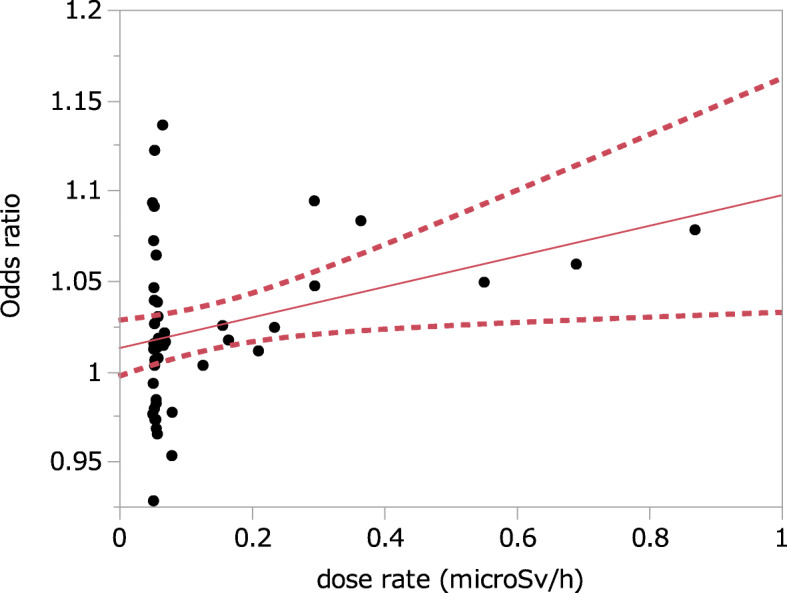
Unrestricted linear regression analysis between dose rates and odds ratios in 47 prefectures in Japan shown in the report [1]. Red line showed regression line with 95% confidence intervals (dotted lines). R^2^ = 0.108 and *p* = 0.0239 for slope factor

According to explanations above, we suggest that authors will clarify the assumptions and rationale in the study.

## Data Availability

Data and material sharing are not applicable to this letter.
